# Essential Infantile Esotropia: A Course of Treatment From Our Experience

**DOI:** 10.3389/fped.2021.695841

**Published:** 2021-07-23

**Authors:** Stefano Pensiero, Laura Diplotti, Marianna Presotto, Luca Ronfani, Egidio Barbi

**Affiliations:** ^1^Department of Ophthalmology, Institute for Maternal and Child Health - IRCCS Burlo Garofolo, Trieste, Italy; ^2^Department of Medical, Surgical and Health Sciences, University of Trieste, Trieste, Italy; ^3^Clinical Epidemiology and Public Health Research Unit, Trieste, Institute for Maternal and Child Health—IRCCS Burlo Garofolo, Trieste, Italy; ^4^Department of Pediatrics, Institute for Maternal and Child Health—IRCCS Burlo Garofolo, Trieste, Italy

**Keywords:** essential infantile esotropia, botulinum toxin chemodenervation, strabismus surgery, stereopsis, refraction, minimally-invasive surgery, protocol, children

## Abstract

**Background:** Essential infantile esotropia (EIE) is the most common type of childhood esotropia. Although its classical approach is surgical, less invasive techniques have been proposed as an adjunct or alternative to traditional surgery. Among them, chemodenervation with botulinum toxin (BT) has been investigated, showing variable and sometimes conflicting results.

**Objectives:** To compare the outcomes of bilateral BT injection and traditional surgery in a pediatric population with EIE in order to optimize and standardize the therapeutic approach. Other purposes are to evaluate whether early intervention may prevent the onset of vertical ocular deviation (which is part of the clinical picture of EIE) and/or influence the development of fine stereopsis, and also to assess changes in refractive status over time among the enrolled population.

**Methods:** A retrospective consecutive cohort study was conducted in 86 children aged 0–48 months who underwent correction of EIE. The primary intervention in naïve subjects was either bilateral BT injection (36 subjects, “BT group”) or strabismus surgery (50 subjects, “surgery group”).

**Results:** Overall, BT chemodenervation (one or two injections) was effective in 13 (36.1%) subjects. With regard to residual deviation angle, the outcomes at least 5 years after the last intervention were overlapping in children receiving initial treatment with either injection or surgery; however, the success rate of primary intervention in the surgery group was higher, and the average number of interventions necessary to achieve orthotropia was smaller. Both early treatment with chemodenervation and surgery at a later age were not found to prevent the onset of vertical ocular deviation, whereas, surprisingly, the percentage of subjects developing fine stereopsis was higher in the surgery group. Finally, with regard to the change in refractive status over time, most of the subjects increased their initial hyperopia, whereas 10% became myopic.

**Conclusions:** Our data suggest that a single bilateral BT injection by age 2 years should be considered as the first-line treatment of EIE without vertical component; whereas, traditional surgery should be considered as the first-line treatment for all other cases and in subjects unresponsive to primary single BT injection.

## Introduction

Essential infantile esotropia (EIE) is the most common type of childhood esodeviation. It typically occurs during the first 6 months of life and is characterized by a constant, large angle strabismus, in neurologically normal and otherwise healthy children. It may be associated with small-to-moderate hyperopia, inferior oblique muscle (IO) hyperfunction, dissociated vertical deviation (DVD), absent or reduced binocular single vision (BSV), sometimes mild amblyopia, and/or latent nystagmus (1, 2).

The classic surgical approach to EIE is bilateral medial rectus (MR) muscle recession, if needed, combined with uni- or bilateral lateral rectus (LR) muscle resection (3).

Since the 1980s, chemodenervation with botulinum toxin (BT) has been proposed as an adjunct or alternative to strabismus surgery (4), and it is currently approved by the FDA in patients older than 12 years (5). As BT injection is less invasive than surgery and seems to be associated with viable motor and sensory results, several studies investigated its use in the treatment of infantile strabismus, including EIE (6), with variable results. Aside from methodological differences (in terms of technique, age at first treatment, and/or evaluation of results), inclusion criteria differences may account for the variability of results observed during studies. Indeed, in most of the available studies, the inclusion criteria were very broad (in terms of etiology and associated conditions, amount of deviation angle, cycloplegic refraction, etc.) and what it is usually referred to as “infantile” or “congenital” esotropia, actually, comprises various strabismic conditions, other than EIE. This may be misleading and bias data interpretation. In this regard, already in 2013, Elliott and Shafiq (7) concluded their review on the management of infantile esotropia stating that it had not been possible to establish the superiority of chemodenervation over traditional surgery, or vice versa and outlined the need for further good quality standardized studies in order to improve the evidence-based treatment approaches.

The aim of this retrospective study is to compare the outcomes of chemodenervation and traditional surgery in a pediatric population with EIE (by evaluating the success rate of primary intervention, the need for reintervention, and, possibly, the number of interventions necessary to achieve acceptable ocular alignment and BSV development) in order to optimize and standardize the therapeutic approach. Other purposes are to evaluate whether early intervention may prevent the onset of vertical ocular deviation (which is part of the clinical picture of EIE) and/or influence the development of fine stereopsis and to assess changes in refractive status over time among the enrolled population.

## Materials and Methods

Medical records of patients who underwent correction of EIE at the Institute for Maternal and Child Health of Trieste *IRCCS Burlo Garofolo* (Trieste, Italy) between 2003 and 2015, with a post-operative follow-up of at least 5 years, were investigated. The research was approved by the Institutional Review Board of the IRCCS Burlo Garofolo and adheres to the tenets of the Declaration of Helsinki.

EIE was defined as constant, stable, large-angle [>30 prism diopters (PD)] esotropia, occurring before age 6 months in neurologically normal and otherwise healthy children (2, 8).

To differentiate EIE from early-onset accommodative esotropia, full cycloplegic correction was prescribed in all subjects older than 7 months, with mono- or bilateral spherical equivalent (SE) refraction > +2.50 diopters (D); if no change in the strabismic angle was found after optical correction, patients were diagnosed with EIE and enrolled in the study (2).

Inclusion criteria comprised also cycloplegic SE refraction between 0 and +5.00 D at the time of the first intervention. The refractive cutoff values used take into account the changes in refractive error in aging children with EIE described by Birch et al. (9), and the research of Lee et al. (10), who found that preoperative hyperopia < +5.00 D does not affect the outcome of EIE surgery.

Preterm infants, small for date infants, subjects with ophthalmological and/or neurological disorders other than latent nystagmus (e.g., restrictive or paralytic ocular motility disorders, congenital ocular malformations, deprivation amblyopia, perinatal hypoxic-ischemic injuries, perinatal, and post-natal neurological disorders, and development delay) were excluded from the study.

The primary intervention in naïve patients was either BT chemodenervation (BT group) or traditional strabismus surgery (surgery group).

Chemodenervation involves the transconjunctival injection of 10 units of botulinum type A toxin (Dysport®, Ipsen SpA, Milano, Italy) in both MR, without conjunctival incision and without electromyographic guidance (11, 12). The technique consists of pulling the eye into an abducted position, grasping the MR transconjunctivally with forceps, and then injecting BT using a 30-G needle through the nasal conjunctiva at about 8–10 mm from the limbus, targeted at the belly of the muscle (13).

Surgical correction of EIE consisted of standard bilateral MR recession, if needed, associated with uni- or bilateral LR resection. The amount of surgery was calculated according to the following rule: 1 mm MR recession for every 3 PD of deviation angle (up to a maximum of 6 mm recession), eventually combined with 1 mm ML resection for every 3 PD (up to 5 mm resection). Thus, we performed symmetrical bilateral surgery. This approach results from the adaptation of the nomograms proposed in the scientific literature to our personal experience and is superimposable to the guidelines given by Taylor et al. (14). Concomitant or deferred, bilateral recession with anterior transposition of the IO was performed in subjects with V-pattern strabismus, and uni- or bilateral superior rectus muscle (SR) recession was performed in subjects with clinically relevant DVD.

The outcome was classified as follows: *complete success* (defined as orthotropy, i.e., final deviation angle ≤8 PD); *partial success* (defined as the final deviation angle reduced to at least half of its original amount, but still >8 PD); or *failure* (in all other cases).

In the BT group, all patients underwent primary chemodenervation. If the success at 6 months was only partial, the treatment was repeated 6 to 9 months after the first injection. All subjects with the unsuccessful outcome and all subjects who, after the second injection, presented only a partial success underwent subsequent surgical correction of strabismus, between age 2 and 4 years.

In the surgery group, all patients underwent primary horizontal muscles surgery, eventually associated with bilateral IO recession. If the success was only partial, or subsequent ocular vertical deviation occurred (either due to IO or SR hyperfunction), subjects underwent an additional tailored vertical and/or horizontal surgical operation, at least 12 months after the previous treatment.

Data collected included: gender, type of procedure and age at the time of each intervention, pre- and post-operative near and, if possible (according to child's age and compliance), distant angles of deviation; pre- and post-operative cycloplegic refraction, and postoperative presence of fine stereoacuity [Titmus test <160 seconds of arc (″)] (15). Pre-operative data refer to the day before the first intervention, post-operative data to the last available follow-up, at least 5 years after the last intervention.

Descriptive statistics were used to report the results. Categorical variables were presented as numbers and percentages, continuous variables as mean value with SD. The Wilcoxon–Mann–Whitney test was used to assess the difference in the distribution of a continuous variable across two groups of a categorical variable. The Chi-square test was used to determine the association between two categorical variables. The Fisher's exact test was employed instead of Chi-square test when sample sizes were small. Statistical significance was defined as *p* < 0.05.

The mean difference (MD) and the odds ratio (OR), with 95% CI, were used to compare the continuous and the dichotomous outcome data, respectively. All statistical analyses were conducted using the SAS software, Version 9.4 (SAS Institute Inc., Cary, NC, USA).

As it was not possible to assess the distant angle of deviation in all enrolled subjects, only the near angle of deviation was taken into consideration for the statistical analysis.

## Results

Overall, 86 children, 48 males, and 38 females, were enrolled in this study. Their clinical characteristics are described in Tables 1–3.

**Table 1:** Ophthalmologic characteristics of the population enrolled in the “*BT group*”.

		**1st int**.		**2nd int**.		**3rd int**.		**4th int**.		**Baseline ^a^**						**Last follow-up ^b^**						
**ID**	**Gen**	**Age (y)**	**Int.**	**Age (y)**	**Int.**	**Age (y)**	**Int.**	**Age (y)**	**Int.**	**Near α (PD) ^c^**	**Dist. α (PD) ^c^**	**RE SE (D)**	**LE SE (D)**	**RE Cyl (D)**	**LE Cyl (D)**	**Near α (PD) ^c^**	**Dist. α (PD) ^c^**	**RE SE (D)**	**LE SE (D)**	**RE Cyl (D)**	**LE Cyl (D)**	**Stereoacuity (“) ^d^**
1	F	0	BT							35		3.00	3.88	1.00	0.75	14	14	2.75	3.13	0.50	0.75	
2	F	1	BT							30		2.38	1.50	0.75	1.00	0	0	1.25	0.25	0.50	0.50	
3	F	1	BT							30		4.50	4.50	1.00	1.00	0	0	−0.25	−0.13	1.00	0.75	
4	M	1	BT							30		1.88	1.00	0.75	0.50	0	0	2.38	2.13	0.75	1.25	
5	F	1	BT							45		1.25	1.50			4	0	0.50	0.75	0.50	0.50	
6	M	1	BT							45		1.75	1.75	2.50	2.50	0	0	2.38	2.50	2.75	3.00	
7	M	1	BT							40		1.38	1.25	0.75	0.50	20	18	0.75	1.13		0.75	
8	F	1	BT							30		2.50	2.50			14	12	3.25	3.00			
9	F	2	BT							35		4.38	3.75	1.25	1.00	2	0	5.13	4.88	2.25	1.75	
10	M	1	BT	2	BT					35		3.88	3.88	0.75	0.75	16	12	5.00	4.75	0.50	1.50	
11	M	1	BT	2	BT					40		1.00	0.88	1.00	0.75	0	0	−0.88	−0.25	2.25	1.50	
12	M	0	BT	1	BT	2	H	3	H	30		0.88	0.38	0.75	0.75	4	0	−1.75	−1.63	1.00	1.25	
13	M	0	BT	1	BT	2	H	3	H	40		1.00	0.50		1.00	14	12	2.00	2.00		0.50	
14	F	1	BT	2	BT	2	H	5	H+IO	40		2.88	2.50	2.25	2.50	4	−6	2.50	2.88	2.50	2.25	140
15	F	0	BT	1	BT	3	H+IO			70		1.88	1.75	0.75	1.00	18	4	3.13	2.88	2.25	1.75	
16	M	0	BT	1	BT	2	H+IO			70		1.75	1.50	0.50	0.50	6	12	2.50	2.00		0.50	
17	M	1	BT	2	BT	4	H+IO			35		1.13	1.25	1.25		6	4	2.00	1.38	2.00	1.25	
18	M	1	BT	2	BT	4	H+IO			25		1.88	0.00	1.25		12	10	0.25	0.50			
19	M	1	BT	2	BT	4	H+IO			45		3.50	4.00	1.00	1.00	−6	−8	1.50	3.25	1.00	0.50	
20	M	1	BT	2	BT	3	H+IO			40		2.00	1.75	0.50		16	16	2.50	2.75			
21	F	1	BT	2	BT	2	H+IO			50		0.50	0.50	1.00	1.00	6	2	1.75	1.50	1.00	0.50	
22	M	1	BT	4	H					40		4.25	4.75			0	0	7.13	6.38	2.75	2.75	
23	M	1	BT	3	H					70		2.75	2.50	1.50	2.00	18	6	4.88	6.00	0.75		
24	F	1	BT	2	H					35		1.75	1.75			4	4	3.38	3.63	0.75	1.25	
25	F	1	BT	3	H					30		4.00	4.50	1.50	0.50	10	10	4.75	4.75	1.50	1.50	
26	M	1	BT	4	H	7	H			70		1.88	0.75	0.75	1.00	4	4	1.75	0.88	1.00	0.75	
27	F	0	BT	3	H	5	H+IO			40		2.00	1.50			4	4	3.00	3.00			
28	M	0	BT	2	H	4	H+IO			40		2.25	1.38	2.50	1.25	6	8	1.75	1.13	2.50	1.25	
29	M	1	BT	3	H	6	H+IO	10	SR	35		4.00	3.25	1.00	0.50	10	8	5.75	5.50	1.00	1.00	140
30	F	1	BT	2	IO	7	H			30		2.50	2.50		0.50	−8	−10	1.50	2.00	0.50	0.50	
31	F	2	BT	5	IO	12	SR			50		2.00	2.00			4	−2	3.25	3.75		0.50	60
32	F	0	BT	3	H+IO	4	H			35		3.25	3.75			0	0	4.00	4.25		0.50	60
33	F	1	BT	2	H+IO	10	SR			40		1.00	1.13	2.00	2.25	16	−4	−1.63	−1.13	1.75	3.25	
34	M	1	BT	5	H+IO					30		4.75	5.00	1.50	1.50	2	2	5.75	4.63	1.50	1.75	
35	M	1	BT	2	H+IO					40		3.75	3.75	0.50	1.00	25	2	4.25	4.38	0.50	0.75	
36	M	1	BT	4	H+IO					45		1.00	2.00	0.50	0.50	0	0	1.88	2.50	0.75	1.00	

*ID, identification number; Gen, gender; y, years; Int., intervention; Dist., distance; α, angle of deviation; PD, prism diopters; D, diopters; RE, right eye; LE, left eye; SE, spherical equivalent; Cyl, cylinder; ″, seconds of arc; M, male, F, female; BT, chemodenervation with botulinum toxin; H, horizontal muscle surgery; IO, inferior oblique muscle recession; SR, superior rectus muscle recession*.

a *The day before the first intervention;*

b *at least 5 years after the last intervention;*

c *positive numbers refer to esodeviations; negative to exodeviations;*

d *Titmus test <160″*.

**Table 2:** Ophthalmologic characteristics of the population enrolled in the “*surgery group*”.

		**1st int**.		**2nd int**.		**3rd int**.		**4th int**.		**Baseline ^a^**						**Last follow-up ^b^**						
**ID**	**Gen**	**Age (y)**	**Int.**	**Age (y)**	**Int.**	**Age (y)**	**Int.**	**Age (y)**	**Int.**	**Near α (PD) ^c^**	**Dist. α (PD) ^c^**	**RE SE (D)**	**LE SE (D)**	**RE Cyl (D)**	**LE Cyl (D)**	**Near α (PD) ^c^**	**Dist. α (PD) ^c^**	**RE SE (D)**	**LE SE (D)**	**RE Cyl (D)**	**LE Cyl (D)**	**Stereoacuity (“) ^d^**
37	F	2	H							30		4.75	5.00	2.50	3.00	20	14	5.00	5.25	3.00	3.50	
38	M	2	H							35		2.13	1.63	1.25	1.25	0	−8	3.25	3.13	2.50	1.75	
39	M	3	H							40	30	2.00	2.50			8	8	1.25	3.50	0.50	1.50	60
40	M	3	H							40	30	3.75	3.00	2.50	2.00	−4	−6	5.38	4.13	3.75	2.25	
41	M	3	H							35	30	2.50	3.00			−2	−6	4.13	4.13	1.25	3.75	140
42	M	3	H							35	35	0.75	0.75			14	16	0.25	0.25		0.50	
43	M	3	H							35	35	4.50	4.38	0.50	0.75	14	4	5.00	5.00		0.50	
44	F	3	H							35		3.75	3.25	1.50	1.00	2	2	4.88	4.25	1.75	0.50	
45	F	3	H							35	30	2.50	2.75		0.50	4	2	3.00	3.00	0.50	0.50	100
46	M	3	H							45		3.00	3.00	3.00	1.00	16	14	3.75	3.25	2.50	1.50	
47	F	3	H							30		3.50	3.75	0.50	0.50	4	6	3.25	2.75	1.00		
48	M	3	H							35	30	4.75	5.00	0.50	0.50	0	−6	5.25	5.75	1.00		
49	F	3	H							30		2.25	4.63	0.50	1.25	0	0	4.00	6.13		1.25	
50	M	3	H							40		3.88	5.00	0.75		0	0	4.75	5.25	1.50	1.00	
51	F	3	H							35	30	3.75	3.00			6	0	6.50	6.00	1.00	1.00	
52	F	3	H							40	35	1.13	0.50	0.75		6	0	0.50	−0.25		0.50	
53	F	3	H							30		2.25	2.50	1.00		6	6	1.00	2.38	2.00	0.75	
54	M	3	H							30		2.00	2.00			0	0	2.25	2.00	0.50	0.50	40
55	F	3	H							30	30	4.50	4.63	0.50	0.75	0	0	4.88	5.63	1.75	1.75	
56	M	4	H							35	35	3.38	2.88	1.75	1.25	0	−2	4.50	3.88	2.00	1.75	
57	M	4	H							35	35	3.50	4.25	0.50	0.50	2	0	4.50	5.25			140
58	F	4	H							35	35	1.00	1.00	0.50	1.00	10	10	1.25	2.50	1.00	0.50	
59	F	4	H							40	40	1.25	1.00			4	0	1.50	1.50	0.50		
60	M	4	H							35		4.38	3.88	1.25	1.75	4	2	4.50	4.00	2.00	2.00	
61	M	4	H							35	30	2.00	2.00			12	2	2.50	2.75			
62	F	4	H							30	30	2.25	2.13	3.00	2.75	−2	−4	−0.63	−2.38	2.75	2.75	
63	M	2	H	3	H					30		2.00	1.75	0.50		6	2	1.50	0.50			
64	F	2	H	10	H					30	30	2.88	2.50	0.75		0	0	0.63	0.00	1.25		
65	M	3	H	5	H					30		2.50	2.75	1.50	1.50	4	0	0.38	1.25	1.75	1.50	
66	F	4	H	9	H					35	35	4.00	4.00	1.00	1.50	14	14	4.38	4.63	2.75	3.25	
67	M	4	H	8	H					40	35	1.38	1.88	0.75	1.25	6	4	3.00	3.38	1.00	1.75	140
68	M	3	H	4	H	10	IO			40		4.75	4.88	0.50	0.75	−2	−4	5.25	5.00	0.50	0.50	140
69	F	2	H	7	IO					40		1.25	1.38		0.75	0	0	−2.50	−2.50		1.50	140
70	F	2	H	4	IO	9	H			35	30	4.50	4.50	2.00	2.50	25	20	4.00	5.13	2.50	2.75	
71	M	2	H	9	H+IO					40		2.25	1.63	1.00	0.75	6	0	2.75	1.88	1.00	0.75	
72	F	3	H	6	H+IO					35	30	1.63	1.63	1.25	0.75	4	4	3.38	2.38	1.25	2.25	
73	F	3	H	5	H+IO					35		4.00	3.75	1.50	1.50	−10	−4	4.50	4.63	1.50	1.25	
74	M	4	H	5	H+IO					50	45	0.25	1.00	2.00	1.00	18	16	−1.75	−3.00	0.50	1.00	
75	F	3	H+IO							45		4.00	4.38	0.50	1.25	16	10	5.00	4.75	1.00	1.00	
76	M	3	H+IO							40		3.50	3.50	2.00	2.00	−4	−2	5.13	5.63	1.25	1.25	
77	F	3	H+IO							45		2.38	2.88	0.75	1.25	20	14	2.25	2.00	1.50	1.00	
78	M	4	H+IO							40	30	1.88	1.50	0.75	1.00	10	6	2.50	3.00	1.00	2.00	100
79	M	2	H+IO	4	H					40		2.75	3.25	2.50	2.50	14	14	3.50	2.88	2.50	2.25	
80	M	3	H+IO	9	H					30	30	1.50	1.50	0.50		6	8	0.75	−2.75			80
81	M	3	H+IO	4	H					50		0.75	0.63	1.00	0.75	−6	−2	−2.50	−3.25	1.50	1.00	140
82	F	3	H+IO	4	H					30	30	0.25	0.38	0.50	0.75	14	12	1.63	1.63	0.75	0.75	
83	M	4	H+IO	6	H					30		1.63	1.00	1.25	1.00	8	8	0.25	1.38		1.25	
84	F	4	H+IO	6	H					35	30	2.50	2.38	1.00	0.75	10	12	2.75	2.75	2.50	2.00	
85	M	4	H+IO	4	H					35	35	3.13	3.50	1.25	2.00	−8	−8	3.38	4.00	1.25	2.00	140
86	M	4	H+IO	13	SR					40	30	1.00	0.50			10	8	−2.75	−1.63	0.50	0.75	140

*ID, identification number; Gen, gender; y, years; Int., intervention; Dist., distance; α, angle of deviation; PD, prism diopters; D, diopters; RE, right eye; LE, left eye; SE, spherical equivalent; Cyl, cylinder; ″, seconds of arc; M, male, F, female; BT: chemodenervation with botulinum toxin; H: horizontal muscle surgery; IO: inferior oblique muscle recession; SR, superior rectus muscle recession*.

a *The day before the first intervention;*

b *at least 5 years after the last intervention;*

c *positive numbers refer to esodeviations; negative to exodeviations;*

d *Titmus test <160″*.

**Table 3:** Comparison of the baseline characteristics between the “*BT group*” and the “*surgery group*”.

	**BT group (*n* = 36)**	**Surgery group (*n* = 50)**	***p*-value**	**Statistical analysis**
Sex (males/females)	20/16	28/22	0.97	Chi-square
Age (years)	1 ± 1	3 ± 1	<0.00001	Wilcoxon–Mann–Whitney
Near α (PD)	40.83 ± 12.04	36.20 ± 5.21	0.12	Wilcoxon–Mann–Whitney
Distance α (PD)	N.A.	32.50 ± 3.73		
Right eye spherical equivalent (D)	2.40 ± 1.19	2.64 ± 1.27	0.52	Wilcoxon–Mann–Whitney
Left eye spherical equivalent (D)	2.25 ± 1.39	2.69 ± 1.38	0.77	Wilcoxon–Mann–Whitney

*α, angle of deviation; PD, prism diopters; D, diopters; N.A., not available*.

*Data are expressed as number or mean ± SD*.

In the BT group (Table 1), 36 (41.9%) naïve patients not presenting with vertical ocular deviation, who had undergone chemodenervation before age 2 years (eight aged between 8 and 10 months, 26 aged between 12 and 24 months, and two older than 24 months), were enrolled. The outcome at 6 months was a complete success in 11 children (30.6%; one younger than 12 months), a partial success in 12 (33.3%; four younger than 12 months), and a failure in 13 (36.1%; three younger than 12 months). The 12 subjects presenting only a partial response to chemodenervation, underwent a second BT injection, with favorable outcomes in two (16.7%, none younger than 12 months, and none having fine stereopsis at the last follow-up). The remaining 10 subjects, and the 13 subjects whose deviation angle did not improve after the primary treatment, underwent horizontal muscles surgery.

At baseline, no patients in the BT group presented vertical ocular deviation. Afterward, 18 patients (50%; two with post-injection favorable outcome, eight with the partial outcome, and eight with the unsuccessful outcome) developed IO hyperfunction, and three (8.3%) developed also SR hyperfunction, requiring vertical muscle surgery.

The mean number of interventions performed per patient in the BT group was 2.36 (85 operations on 36 patients). Overall, 13 subjects (36.1%) reached orthotropy in response to chemodenervation and did not require horizontal muscle surgery. Subsequently, three of them had an increase in the strabismic angle that was not corrected. None of these three children ever reached fine stereopsis.

In the surgery group (Table 2), 50 (58.1%) naïve subjects who had undergone horizontal muscles surgery between age 2 and 4 years were enrolled; 12 (24%) of them presented with V pattern strabismus at the time of treatment and had undergone concomitant bilateral IO recession.

Post-operatively, out of 38 children who underwent horizontal surgery alone, 27 (71.1%) reached orthotropy and 11 (28.9%) presented a residual deviation angle requiring further horizontal surgery.

Afterward, seven patients (18.4%, one with favorable outcome and six with partial outcome) developed IO hyperfunction requiring vertical surgery, alone or in combination with further horizontal surgery. Out of 12 subjects who underwent horizontal surgery associated with bilateral IO recession, five (41.6%) reached orthotropy and seven (58.3%) presented a residual deviation angle requiring further horizontal surgery. Afterward, a patient with a favorable outcome (8.3%) developed SR hyperfunction requiring vertical surgery.

Overall, 19 children in the surgery group (38.0%; 12 pre- and seven post-operatively) presented IO hyperfunction, and one (2.0%) developed also SR hyperfunction, requiring vertical surgery.

The mean number of interventions performed per patient in the surgery group was 1.44 (72 operations on 50 patients): 1.4 in naïve subjects undergoing horizontal surgery alone (52 operations on 38 patients), and 1.6 in naïve subjects undergoing both horizontal and vertical surgery (20 operations on 12 patients).

No statistically significant differences in the baseline characteristics were found between the two groups, except for age at the first intervention (as per the design of study, Table 3).

With regard to the horizontal component of strabismus, the success rate of primary intervention was higher in the surgery group (71.1%) than in the BT group (30.6%), and the difference was found to be statistically significant (*p* = 0.0005). No significant difference was found between the two groups in the occurrence of IO hyperfunction (*p* = 0.27; Table 4), whereas the postoperative occurrence of vertical squint was higher in the BT group (*p* = 0.004; Table 4). Of note, among patients of both groups who underwent correction of the horizontal component alone, the occurrence of IO hyperfunction was significantly higher in those who did not reach a satisfactory outcome than in the others (69.6 vs. 15.4% in the TB group, *p* = 0.001 and 54.5 vs. 3.7% in the surgery group, *p* = 0.0002, respectively).

**Table 4:** Comparison of the main study outcomes between the “*BT group*” and the “*surgery group*”.

	**BT group (*n* = 36)**	**Surgery group (*n* = 50)**	***p*-value**	**Statistical analysis**	**OR**	**MD**	**95% CI**
Success of primary intervention	11 (30.6)^a^	32 (64.0)	0.002	Chi-square	0.25		0.1,0.62
Number of interventions per patient	2.36 ± 0.99^b^	1.44 ± 0.58^c^	<0.0001	Wilcoxon–Mann–Whitney		0.92	0.58, 1.26
Near α at the last follow-up (PD)	6.81 ± 7.69	5.70 ± 7.68	0.73	Wilcoxon–Mann–Whitney		1.11	−2.23, 4.45
Distance α at the last follow-up (PD)	3.72 ± 6.61	3.72 ± 7.02	0.87	Wilcoxon–Mann–Whitney		0	−3.0, 3.0
Occurrence of IO muscle hyperfunction							
- preoperative	0 (0)	12 (24.0)					
- postoperative	18 (50.0)	7 (14.0)	0.004	Chi-square	4.43		1.55, 12.64
- overall	18 (50.0)	19 (38.0)	0.27	Chi-square	1.63		0.69, 3.89
Development of fine stereopsis	4 (11.1)	13 (26.0)	0.11	Fisher	0.36		0.11, 1.2

*α, angle of deviation; PD, prism diopters; IO, inferior oblique; OR, odds ratio; MD, mean difference*.

a *12 subjects presenting only a partial response to chemodenervation, underwent a second BT injection, with favorable outcome in 2 (16.7%);*

b *85 operations on 36 patients;*

c *72 operations on 50 patients*.

*Data are expressed as number of patients (%) or mean ± SD*.

A statistically significant difference was found between the mean number of interventions per patient performed in the two groups (*p* < 0.0001; Table 4), with patients more frequently achieving orthotropia after a single intervention in the surgery group.

Overall, the mean near and distant angles of deviation at baseline and 5 years after the last intervention were 38.14 ± 8.98 PD and 32.50 ± 3.73 PD, and 6.16 ± 7.66 PD and 3.72 ± 6.81 PD, respectively. In the BT group, the mean near angle of deviation at baseline was 40.83 ± 12.04 PD, and the mean near and distant angles of deviation 5 years after the last intervention were 6.81 ± 7.69 PD and 3.72 ± 6.61 PD. In the surgery group, the mean near and distant angles of deviation at baseline and 5 years after the last intervention were 36.20 ± 5.21 PD and 32.50 ± 3.73 PD, and 5.70 ± 7.68 PD and 3.72 ± 7.02 PD, respectively. The mean near-distance disparity at the last follow-up was +2.44 ± 4.57 PD (+3.08 ± 5.86 PD in the BT group and +1.98 ± 3.33 PD in the surgery group), the angle of misalignment at near exceeding that at distance by 10 PD or more in only seven children (five in the BT group and two in the surgery group). In six subjects of the BT group (16.7%) and nine of the surgery group (18.0%), the final outcome was not satisfactory, with a final strabismus angle >10 DP, both at near and distant. No statistically significant difference in the mean angle deviation at both near and distant was found between patients in the BT and in the surgery groups at the last follow-up (*p* = 0.73 and 0.87, respectively; Table 4). A difference in the mean near angle deviation at baseline was found between patients in the BT group who benefited (35.9 ± 5.8 PD) and who did not benefit (43.0 ± 13.5 PD) from chemodenervation, but it did not reach statistical significance (*p* = 0.05).

With regard to cycloplegic refraction, the mean change in the SE during the study period was: +0.06 ± 1.49 D in the right eye and +0.09 ± 1.63 D in the left eye. During the observation time, 19 (22.1%) subjects become less hyperopic; nine (10.5%) with baseline mild hyperopia became myopic; and the remaining 58 (67.4%) maintained or, much more often, increased their hyperopia. The mean change in the refractive cylinder during the study period was +0.21 ± 0.68 D in the right eye and +0.30 ± 0.74 D in the left eye. Except for two cases presenting an increase in the cylinder >1.50 D, astigmatism showed a mild slow change over time.

At the last follow-up, 17 (20%) children had fine stereopsis (<160″). Stereoacuity was 140″ in 10 subjects (two in the BT group, both operated after 12 months of age, and eight in the surgery group), between 40″ and 100″ in seven subjects (two in the BT group: one operated before 12 months of age and the other at age 2 years; and five in the surgery group: four operated at age 3 years and one at age 4 years). All of these children presented a strabismic angle <10 PD (either esotropia or exotropia) at the last follow-up. No statistically significant difference in the development of fine stereopsis was found between patients in the BT and in the surgery group (*p* = 0.11; Table 4).

## Discussion

A growing body of literature has investigated the role of BT injections in the treatment of EIE, with variable and sometimes conflicting outcomes. Apart from methodological differences, inclusion criteria differences may account for the variability of results observed across studies. In fact, except for the study by Campos et al. (enrolling only children with EIE), most of the available studies focused on patients with “congenital” or, more often, “infantile” esotropia, generic conditions including various types of strabismus, other than EIE (6, 12, 16, 17). This may be misleading and bias data interpretation; hence, in this study, only children with EIE were enrolled.

According to our study, transconjunctival bilateral BT injection is effective in 30.6% of subjects and represents a valuable alternative to surgery in the treatment of EIE, especially in subjects with smaller ocular deviations. De Alba Campomanes et al. (18) and Mayet et al. (19) got similar results (chemodenervation success rate of 36 and 37%, respectively), though in their studies the outcomes were obtained after three BT injections and in shorter follow-up periods (20 and 6 months, respectively). Baggesen & Arnljot (12) and Solebo et al. (17) also came to similar conclusions; however, their results cannot be easily compared to ours. Indeed, both studies enrolled subjects aged up to 15 years at the time of primary intervention, and presenting with various types of strabismus, other than EIE (which overall accounted for less than two dozen cases). Moreover, the success of chemodenervation was defined, in the first study (12), as an important reduction in the baseline strabismic angle (not always leading to orthotropy), obtained after one to three injections; and, in the second (17), as 4 months postoperative ocular deviation <11 PD. Our data are also partially consistent with Issaho et al. (6), as we both found that subjects with smaller ocular deviations benefit most from chemodenervation, though in our series the difference, although present, was not significant. According to Issaho's review with meta-analysis, BT injection in patients with congenital esotropia had better outcomes for the ocular deviations <30 PD, which, by definition, cannot be classified as EIE. Our study, on the contrary, only included patients with ocular deviations >30 PD.

In our series, children operated before the year of life did not reach better results than children operated at older ages. Indeed, none of the eight subjects treated with BT injection between age 8 and 10 months maintained orthotropy at the last follow-up. Unlike our study, data in the literature suggest that early EIE correction is associated with better outcomes, though there is no clear consensus on the optimal timing of surgery. Issaho (6) and Singh (20) recommended, respectively, BT injection and EIE surgery, before 12 months of life; Wong (21) concluded his review stating that the EIE surgery has better results when performed at or before 10 months of age, whereas Campos et al. (16) found that the BT injection is associated with favorable results only in infants treated by age 7 months.

With regard to residual deviation angle, in our series, the long-term final outcomes of bilateral chemodenervation and traditional surgery in naïve children with EIE were overlapping (with a final average near deviation angle of +7 PD and +6 PD, respectively), though, in the surgery group, the success rate of primary intervention was higher (64.0 vs. 30.6%; Table 4), and the average number of interventions necessary to achieve acceptable ocular alignment smaller (1.44 vs. 2.36). The difference between the number of interventions per patient performed in the two groups could be related to the different postoperative occurrence of vertical strabismus (50 and 18.4%, respectively, in the BT and surgery group), and, ultimately, to the different age at the time of first intervention. Indeed, IO overaction is not usually present before age 1 year (22), and we did not perform BT injection in patients with either baseline ocular vertical deviation or previous surgical correction of ocular vertical deviation, whereas if needed, we performed concomitant horizontal and vertical strabismus surgery in naïve patients of surgery group. As a confirmation of this, no statistically significant difference was found between the two groups in the overall occurrence of IO hyperfunction (50 and 38.0%, respectively).

In our series, only four children, three in the BT group and one in the surgery group, postoperatively developed SR hyperfunction. All of them had already undergone IO surgery. The sample is too small to draw statistically significant conclusions, nevertheless, our data seem in contrast to Black's findings (23), which suggest that recession with anterior transposition of the IO reduces the risk of subsequent DVD development.

Data in the literature suggest that stereopsis is not present at birth and typically develops during the first years of life: increasing from 800″ at age 4 months to 110″ by age 12 months and approaching adult levels by age 24 months, though it continues improving till age 8–9 years (24–26). More recently, Li et al. (27) proposed that stereopsis may still be enhanced from coarse (>200″) to fine (<200″) [anatomical–functional classification first proposed by Wilcox & Allison (15)] in adulthood, by perceptual learning. All the literature agrees that ocular alignment is one of the conditions necessary for the development of BSV.

In strabismic patients, an inverse relationship has been observed between the sensory outcome and both the amount of the deviation angle (24) and the duration of misalignment (28, 29). Stewart et al. (24) performed one of the largest studies on this topic and reported that subjects with strabismic angle <10 PD develop the best stereopsis, whereas subjects with >25 PD deviation are unlikely to achieve even coarse stereoacuity. A limitation of this study is to have not distinguished between manifest and latent strabismus for deviations <25 DP, making it impossible to assess fine stereopsis in this group. Despite this improvable bias, all patients presented with a stereopsis >160″. Regarding the optimal timing of surgery in relation to sensory outcome, data in the literature agree on the need for early intervention (30, 31). Yagasaki et al. (30) found that only 31.8% of patients operated by age 8 months, and no patient operated at older ages developed stereopsis <200″, whereas Birch and Stager (31) found that 20% of subjects operated by age 8 months vs. only 9% of subjects operated between age 7 and 12 months reached stereopsis <200″.

In our series, 17 subjects developed fine stereopsis (<160″): four in the BT group (out of 36; 11%) and 13 in the surgery group (out of 50; 26%). Among them, a quarter of cases was first operated after age 2 years. These data differ from those reported in the literature and may be explained by the normal development of BSV in children, as already mentioned above (24–27).

Several studies have observed that the benefit of early correction of infantile esotropia for the sensory outcome is balanced by a higher re-operation rate (32, 33) and a higher risk of overtreatment (as some cases might improve spontaneously) (8, 33). Moreover, regardless of the type and timing of intervention, postoperatively, only a few subjects reach more than coarse stereopsis (34). In view of these considerations, early treatment should be preferred only when associated with the chance for postoperative development of fine stereopsis, given the least surgical and anesthesiological risk.

It should be remembered that, even though BT injection and strabismus surgery carry a very low risk of serious complications (35), anesthesiological risks in children should not be underestimated. In 2016, the US Food and Drug Administration issued a safety communication stating that the repeated or prolonged (>3 h) exposure to anesthetics in children younger than 3 years may negatively affect brain development (36, 37). The statement was mainly based on *in vitro* and animal studies, whereas as expected in the absence of a control group, clinical studies seem ambivalent (38).

Glatz et al. (39) reported that the exposure to anesthetics by age 4 years, regardless of the number of exposures, slightly affects the cognitive performance at age 16 years, with cases presenting lower school grades and IQ test scores than age- and sex-matched controls (mean difference: 0.41 and 0.97%, respectively). According to the article, the anesthesiological risk remains unchanged during the first 3 years of life, regardless of the age of subjects at the time of exposure and the number of procedures performed. On the other hand, McCann et al. (40) found no neurodevelopmental differences between 5-year-old children, who, at age 60 weeks, received either general anesthesia or awake-regional anesthesia for inguinal herniorrhaphy; and concluded that 1-h exposure to general anesthesia in early infancy does not seem to affect psychomotor development. Consistently, O'leary (41) and Lee et al. (42) suggested that, in infants, a single exposure to general anesthesia and/or exposures up to 1 h are not associated with detectable risks of long-term neurotoxicity.

In our opinion, and according to the literature, though the real impact of general anesthetics on psychomotor development is still not clear, it appears prudent to avoid excessive exposure in infants.

With regard to the duration of the surgical procedure in our cohort, bilateral BT injections usually lasted 10–15 min, whereas strabismus surgery duration depended on the number of muscles operated (considering that horizontal surgery lasted on average 20–25 min per muscle, whereas vertical surgery 10–15 min per muscle). Clearly, sedation lasted a little longer than operating time.

In view of the above and of our study results, we recommend that a single BT injection in both MR by age 2 years should be considered as the first-line treatment of EIE, in the absence of V-pattern strabismus (with predictable success in one-third of cases, about half of whom will subsequently develop IO hyperfunction requiring surgical correction), whereas traditional surgery should be reserved for all other cases, including those unresponsive to primary single chemodenervation. As IO overaction usually develops after age 1 year, surgery should be performed between age 2 and 4 years in order to possibly correct both defects in a single operation. The development of DVD is uncommon, usually occurs later in life, and if necessary, will be operated accordingly.

Concerning safety issues, even though it has been reported that BT injection may cause potential complications, such as ptosis, vertical deviation, subconjunctival or retrobulbar hemorrhage, scleral penetration, and systemic allergic reaction (43–47), our patients well tolerated the procedure, not presenting with serious or permanent complications, but only transient ptosis (40% of cases).

Finally, with regard to refraction, according to data in the literature, children with EIE present a different pattern of refractive development compared to normal children; indeed, most of them present with low-to-moderate hypermetropia before age 6 months, which either remains stable or increases till age 5 years. After age 5 years, similar to normal cohorts, children with EIE show myopic shifts (9).

Khan (48), analyzing 113 children with EIE, found that subjects presented with mild hypermetropia (mean cycloplegic SE: +1.73 D) and mild astigmatism (mean cycloplegic cylinder: 0.39 D) before age 2 years, which remained stable up to age 10 years (with no statistically significant mean decrease in SE of 0.43 D and no statistically significant mean increase in cylinder of 0.32 D). Birch et al. (9) analyzed 143 children with EIE and found that 55% of subjects had a cycloplegic SE lower than +3.00 D in the first year of life. Hypermetropia presented an initial slight increase up to age 7 years and a subsequent decrease by about 0.5 D per year, with a mean final refraction of +1.00 ± 2.25 D at age 12 years.

Differently from what has been reported in the literature, in our series 67.4% of children maintained or, much more often, increased their hyperopia, 22.1% become less hyperopic, and 10.5% became myopic, demonstrating that the increasing prevalence of myopia among young generations also affects subjects with EIE. According to the data in the literature, also in our cohort, astigmatism showed only mild slow changes over time.

## Conclusions

Chemodenervation is a minimally invasive procedure, requiring only short-term anesthesia, suitable to be performed also at a very young age. Our data show that a single transconjunctival BT injection in both MR before age 2 years, when the vertical component is very rare, is effective in about one-third of cases, while treatment repetition does not show useful. Considering that families frequently advocate for early intervention, both to improve the cosmetic appearance of the child and to ease the ophthalmic management (compliance with patching is often poor in young children), it appears appropriate to consider BT injection as the first-line treatment of EIE without vertical component in children up to age 2 years. Surgery should be considered as the first-line treatment in subjects with concomitant vertical ocular deviations, subjects older than age 2 years, and subjects unresponsive to single injection. As the vertical component usually develops later, surgery should be performed between age 2 and 4 years in order to possibly correct both defects in a single operation, thus reducing the number of required anesthesiological procedures.

Both early treatment with BT injection and surgery at a later age were not found to prevent the onset of vertical ocular deviation, which is part of the clinical picture of EIE, whereas, surprisingly, the percentage of subjects developing fine stereopsis was higher in the surgery group. With regard to residual deviation angle, the long-term final outcomes were overlapping in children receiving initial treatment with either early BT injection or strabismus surgery at a later age, though the success rate of primary intervention in the surgery group was higher and the average number of interventions necessary to achieve acceptable ocular alignment smaller. In this respect, it should be remembered that the exposure to anesthetics may negatively affect brain development in young children, with a dose–response relationship, regardless of the age at the time of exposure and the number of procedures performed.

Finally, with regard to the change in refractive status over time, most of the patients in our cohort increased their initial hyperopia, whereas 10% became myopic, demonstrating that the increasing prevalence of myopia among young generations also affects subjects with EIE. On the contrary, astigmatism showed only mild slow changes over time.

## Data Availability Statement

The original contributions presented in the study are included in this published article; further inquiries can be directed to the corresponding author.

## Ethics Statement

This research was approved by the Institutional Review Board of the Institute for Maternal and Child Health *IRCCS Burlo Garofolo*, and adheres to the tenets of the Declaration of Helsinki. Proper informed consent for the treatment was obtained prospectively from the parents of all subjects who underwent correction of essential infantile esotropia.

## Author Contributions

SP and EB conceived and designed the study. MP acquired the data. LR performed the statistical analysis. SP and LD drafted the manuscript and the tables. All the authors discussed the results and contributed to the interpretation of the data, had full access to all the data in the study and took responsibility for the integrity of the data, and the accuracy of the data analysis as well as the decision to submit for publication.

## Conflict of Interest

The authors declare that the research was conducted in the absence of any commercial or financial relationships that could be construed as a potential conflict of interest.

## Publisher's Note

All claims expressed in this article are solely those of the authors and do not necessarily represent those of their affiliated organizations, or those of the publisher, the editors and the reviewers. Any product that may be evaluated in this article, or claim that may be made by its manufacturer, is not guaranteed or endorsed by the publisher.

## Abbreviations

′′: seconds of arc
BSV: binocular single vision
BT: botulinum toxin
Cyl: cylinder
D: diopters
Dist.: distance
DVD: dissociated vertical deviation
EIE: essential infantile esotropia
F: female
Gen: gender
H: horizontal muscles surgery
ID: subject identification number
Int.: intervention
IO: inferior oblique muscle
LE: left eye
LR: lateral rectus muscle
M: male
MD: mean difference
MR: medial rectus muscle
OR: odds ratio
PD: prism diopters
RE: right eye
SE: spherical equivalent
SR: superior rectus muscle
α: deviation angle.

## References

[1] Costenbader FD. Infantile esotropia. Trans Am Ophthalmol Soc. (1961) 59:397–429. 13881627 PMC1316420

[2] Wright KW Spiegel PH Thompson LS. Handbook of Pediatric Strabismus and Amblyopia. Springer Ed New York. (2006) 56:200–201. 10.3368/aoj.56.1.200-a 21149150

[3] Kushner BJ Morton GV. A randomized comparison of surgical procedures for infantile esotropia. Am J Ophthalmol. (1984) 98:50–61. 10.1016/0002-9394(84)90188-0 6377903

[4] Scott AB. Botulinum toxin injection into extraocular muscles as an alternative to strabismus surgery. Ophthalmology. (1980) 87:1044–9. 10.1016/S0161-6420(80)35127-0 7243198

[5] BOTOX (onabotulinumtoxin A) Medication Guide. (2011). Available online at: https://www.accessdata.fda.gov/drugsatfda_docs/label/2011/103000s5232lbl.pdf (accessed March 01, 2021).

[6] Issaho DC Carvalho FR de S Tabuse MKU Carrijo-Carvalho LC de Freitas D. The use of botulinum toxin to treat infantile esotropia: a systematic review with meta-analysis. Invest Ophthalmol Vis Sci. (2017) 58:5468–76. 10.1167/iovs.17-22576 29059315

[7] Elliott S Shafiq A. Interventions for infantile esotropia. Cochrane Database Syst Rev. (2013):CD004917. 10.1002/14651858.CD004917.pub3 PMC7388175 23897277

[8] Pediatric Eye Disease Investigator Group. Spontaneous resolution of early-onset esotropia: experience of the Congenital Esotropia Observational Study. Am J Ophthalmol. (2002) 133:109–18. 10.1016/s0002-9394(01)01316-2 11755846

[9] Birch EE Stager DR Wang J O'Connor A. Longitudinal changes in refractive error of children with infantile esotropia. Eye (Lond). (2010) 24:1814–21. 10.1038/eye.2010.129 20930854 PMC3005800

[10] Lee HJ Kim J-A Kim S-J Yu YS. Relation between preoperative hyperopia and surgical outcome in infantile esotropia. Int J Ophthalmol. (2018) 11:1963–7. 10.18240/ijo.2018.12.15 30588431 PMC6288546

[11] Benabent EC García Hermosa P Arrazola MT. Alió y Sanz JL. Botulinum toxin injection without electromyographic assistance. J Pediatr Ophthalmol Strabismus. (2002) 39:231–4. 10.3928/0191-3913-20020701-12 12148557

[12] Baggesen K Arnljot HM. Treatment of congenital esotropia with botulinum toxin type A. Acta Ophthalmol. (2011) 89:484–8. 10.1111/j.1755-3768.2009.01737.x 19878118

[13] Mendonça TFS Cronemberger MF Lopes MCE Nakanami CR Bicas HEA. [Electromyograph assistance and Mendonça's forceps-a comparison between two methods of botulinum toxin A injection into the extraocular muscle]. Arq Bras Oftalmol. (2005) 68:245–9. 10.1590/S0004-27492005000200017 15905949

[14] Taylor D Hoyt CS. Pediatric Ophthalmology and Strabismus. 3rd Ed. Maarssen, Netherlands: Elsevier Gezondheidszorg. (2005).

[15] Wilcox LM Allison RS. Coarse-fine dichotomies in human stereopsis. Vision Res. (2009) 49:2653–65. 10.1016/j.visres.2009.06.004 19520102

[16] Campos EC Schiavi C Bellusci C. Critical age of botulinum toxin treatment in essential infantile esotropia. J Pediatr Ophthalmol Strabismus. (2000) 37:328–32. 10.3928/0191-3913-20001101-05 11392405

[17] Solebo AL Austin A-M Theodorou M Timms C Hancox J Adams GGW. Botulinum toxin chemodenervation for childhood strabismus in England: National and local patterns of practice. PLoS ONE. (2018) 13:e0199074. 10.1371/journal.pone.0199074 29902283 PMC6001959

[18] de Alba Campomanes AG Binenbaum G Campomanes Eguiarte G. Comparison of botulinum toxin with surgery as primary treatment for infantile esotropia. J AAPOS. (2010) 14:111–6. 10.1016/j.jaapos.2009.12.162 20451851

[19] Mayet I Ally N Alli HD Tikly M Williams S. Botulinum neurotoxin injections in essential infantile esotropia-a comparative study with surgery in large-angle deviations. Eye (Lond). (2021). 10.1038/s41433-020-01300-4. [Epub ahead of print]. 33432167 PMC8526699

[20] Singh A Parihar JKS Mishra SK Maggon R Badhani A. Outcome of early surgery in infantile esotropia: Our experience in tertiary care hospital. Med J Armed Forces India. (2017) 73:129–33. 10.1016/j.mjafi.2016.11.006 28924312 PMC5592263

[21] Wong AMF. Timing of surgery for infantile esotropia: sensory and motor outcomes. Can J Ophthalmol. (2008) 43:643–51. 10.3129/i08-115 19020629 PMC5154744

[22] Hoyt CS. Taylor D. Pediatric Ophthalmology and Strabismus. 4th ed, Edinburgh: Saunders/Elsevier (2013).

[23] Black BC. Results of anterior transposition of the inferior oblique muscle in incomitant dissociated vertical deviation. J AAPOS. (1997) 1:83–7. 10.1016/S1091-8531(97)90003-3 10875082

[24] Stewart CE Wallace MP Stephens DA Fielder AR Moseley MJ. MOTAS Cooperative. The effect of amblyopia treatment on stereoacuity. J AAPOS. (2013) 17:166–73. 10.1016/j.jaapos.2012.10.021 23622448

[25] Giaschi D Lo R Narasimhan S Lyons C Wilcox LM. Sparing of coarse stereopsis in stereodeficient children with a history of amblyopia. J Vis. (2013) 13. 10.1167/13.10.17 23986537

[26] Giaschi D Narasimhan S Solski A Harrison E Wilcox LM. On the typical development of stereopsis: fine and coarse processing. Vision Res. (2013) 89:65–71. 10.1016/j.visres.2013.07.011 23891704

[27] Li RW Tran TT Craven AP Leung T-W Chat SW Levi DM. Sharpening coarse-to-fine stereo vision by perceptual learning: asymmetric transfer across the spatial frequency spectrum. R Soc Open Sci. (2016) 3:150523. 10.1098/rsos.150523 26909178 PMC4736933

[28] Birch EE Fawcett S Stager DR. Why does early surgical alignment improve stereoacuity outcomes in infantile esotropia? J AAPOS. (2000) 4:10–4. 10.1016/S1091-8531(00)90005-3 10675865

[29] Çerman E Eraslan M Ögüt MS. The relationship of age when motor alignment is achieved and the subsequent development of stereopsis in infantile esotropia. J AAPOS. (2014) 18:222–5. 10.1016/j.jaapos.2013.12.017 24924272

[30] Yagasaki T Yokoyama Y Tsukui M. Relationship between stereopsis outcome and timing of surgical alignment in infantile esotropia. J AAPOS. (2020) 24:78.e1–78.e5. 10.1016/j.jaapos.2019.12.015 32224285

[31] Birch EE Stager DR. Long-term motor and sensory outcomes after early surgery for infantile esotropia. J AAPOS. (2006) 10:409–13. 10.1016/j.jaapos.2006.06.010 17070474

[32] Trigler L Siatkowski RM. Factors associated with horizontal reoperation in infantile esotropia. J AAPOS. (2002) 6:15–20. 10.1067/mpa.2002.120644 11907474

[33] Simonsz HJ Eijkemans MJC. Predictive value of age, angle, and refraction on rate of reoperation and rate of spontaneous resolution in infantile esotropia. Strabismus. (2010) 18:87–97. 10.3109/09273972.2010.503491 20843185

[34] Simonsz HJ Kolling GH Unnebrink K. Final report of the early vs. late infantile strabismus surgery study (ELISSS), a controlled, prospective, multicenter study. Strabismus. (2005) 13:169–99. 10.1080/09273970500416594 16361188

[35] Wan MJ Hunter DG. Complications of strabismus surgery: incidence and risk factors. Semin Ophthalmol. (2014) 29:421–8. 10.3109/08820538.2014.959190 25325869

[36] U.S. Food & Drug Administration. FDA Drug Safety Communication: FDA review results in new warnings about using general anesthetics and sedation drugs in young children and pregnant women. (2016). Available online at: https://www.fda.gov/drugs/drug-safety-and-availability/fda-drug-safety-communication-fda-review-results-new-warnings-about-using-general-anesthetics-and (accessed March 01, 2021).

[37] U.S. Food & Drug Administration. FDA drug safety communication: FDA approves label changes for use of general anesthetic and sedation drugs in young children. (2017). Available online at: https://www.fda.gov/drugs/drug-safety-and-availability/fda-drug-safety-communication-fda-approves-label-changes-use-general-anesthetic-and-sedation-drugs (accessed October 16, 2020).

[38] Liu X Ji J Zhao GQ. General anesthesia affecting on developing brain: evidence from animal to clinical research. J Anesth. (2020) 34:765–72. 10.1007/s00540-020-02812-9 32601887 PMC7511469

[39] Glatz P Sandin RH Pedersen NL Bonamy A-K Eriksson LI Granath F. Association of anesthesia and surgery during childhood with long-term academic performance. JAMA Pediatr. (2017) 171:e163470. 10.1001/jamapediatrics.2016.3470 27820621

[40] McCann ME de Graaff JC Dorris L Disma N Withington D Bell G. Neurodevelopmental outcome at 5 years of age after general anesthesia or awake-regional anesthesia in infancy (GAS): an international, multicentre, randomized, controlled equivalence trial. Lancet. (2019) 393:664–77. 10.1016/S0140-6736(18)32485-1. 30782342 PMC6500739

[41] O'leary JD. Human Studies of anesthesia-related neurotoxicity in children: a narrative review of recent additions to the clinical literature. Clin Perinatol. (2019) 46:637–45. 10.1016/j.clp.2019.08.001 31653299

[42] Lee JR Loepke AW. Does pediatric anesthesia cause brain damage? - Addressing parental and provider concerns in light of compelling animal studies and seemingly ambivalent human data Korean. J Anesthesiol. (2018) 71:255–73. 10.4097/kja.d.18.00165 PMC6078876 29969889

[43] Rowe F Noonan C. Complications of botulinum toxin a and their adverse effects. Strabismus. (2009) 17:39–142. 10.3109/09273970903303860 20001507

[44] Yang HK Kim DH Hwang J-M. Botulinum toxin injection without electromyographic guidance in consecutive esotropia. PLoS ONE. (2020) 15:e0241588. 10.1371/journal.pone.0241588 33180838 PMC7660504

[45] Pehere N Jalali S Mathai A Naik M Ramesh K. Inadvertent intraocular injection of botulinum toxin A. J Pediatr Ophthalmol Strabismus. (2011) 48:e1–3. 10.3928/01913913-20110118-06 21261223

[46] Liu M Lee HC Hertle RW Ho AC. Retinal detachment from inadvertent intraocular injection of botulinum toxin A. Am J Ophthalmol. (2004) 137:201–2. 10.1016/S0002-9394(03)00837-7 14700677

[47] Agrawal S Singh V Gupta SK Kumar BV. Vitreous hemorrhage following inadvertent intra-ocular injection of botulinum toxin. Oman J Ophthalmol. (2015) 8:79–80. 10.4103/0974-620X.149905 25709290 PMC4333559

[48] Khan AO. Cycloplegic refractions as a function of age in children with infantile esotropia. Binocul Vis Strabismus Q. (2009)24:39–42. 19323648

